# Supplementary material for: Potential function of CbuSPL and gene encoding its interacting protein during flowering in Catalpa bungei
Source: BMC Plant Biol. 2020 Mar 6;20:105. doi: 10.1186/s12870-020-2303-z (PMC7060540; doi:10.1186/s12870-020-2303-z)
Supplement: Supplementary file 1 — Additional file 1: Table S1. The list of the all Primers used in this paper. [file 12870_2020_2303_MOESM1_ESM.docx]

**Table S1 The list of the all Primers used in this paper.**

|  | Gene name | Primer sequence (5’-3’) |
| --- | --- | --- |
| Primers used in homology-basd cloningof SPL and RACE of homologous gene CbuSPL | SPLF | CAGGTGGAGGGTTGTAAGGT |
|  | SPLR | CCAAGACTCAAGGATCGGGT |
|  | 3’RSPLF1 | GCAAAGGTTTTGCCAGCAGTG |
|  | 3'RACE Out | TACCGTCGTTCCACTAGTGATTT |
|  | 3’RSPLF2 | CGCCTTGCTGGCCATAACGAGCGTA |
|  | 3'RACE Inner | CGCGGATCCTCCACTAGTGATTTCACTATAGG |
|  | 5’RSPLR1 | AGAAGAGAGAGAGCACCGGTG |
|  | 5'RACE Out | CATGGCTACATGCTGACAGCCTA |
|  | 5’RSPLR2 | CCTGATATGGAAGTTGATACTTT |
|  | 5'RACE Inner | CGCGGATCCACAGCCTACTGATGATCAGTCGATG |
|  | QCCbuSPLF | ATGCACTGATTGTATGAGGGAG |
|  | QCCbuSPLR | GACACACAGAAAGCAACAACAT |
| Primers used in PCR of cDNA from homologous gene CbuSPL9 and CbuHMGA | CbuSPL9F | ATGGAAAAGGGTTCTTCCTC |
|  | CbuSPL9R | TCAGATAGAGGATTTTGTGAGTTT |
|  | CbuHMGAF | ATGGCGAGCGAAGAAGTACA |
|  | CbuHMGAR | CTACAATGATCCATAAATTAAAAAAC |
| Primers used in overexpression analysis | 121CbuSPL9F | TCTAGAATGGAAAAGGGTTCTTCCTC |
|  | 121CbuSPL9R | CCTAGGTCAGATAGAGGATTTTGTGAGTTT |
|  | 121CbuHMGAF | TCTAGAATGGCGAGCGAAGAAGTACA |
|  | 121CbuHMGAR | CCTAGGCTACAATGATCCATAAATTAAAAAAC |
| Primers used in analysis for the presence of the transgene | CbuSPL9F | GCCATCATTGCGATAAAGGAAA |
|  | CbuSPL9R | ATCCAGACTGAATGCCCACAGG |
|  | CbuHMGAF | GCCATCATTGCGATAAAGGAAA |
|  | CbuHMGAR | ATCCAGACTGAATGCCCACAGG |
| Primers used in subcellular localization analysis | CbuSPL9-GFPF | CCATGGATGGAAAAGGGTTCTTCCTC |
|  | CbuSPL9-GFPR | TCTAGATCAGATAGAGGATTTTGTGAGTTT |
|  | CbuHMGA-GFPF | CCATGGATGGCGAGCGAAGAAGTACA |
|  | CbuHMGA-GFPR | TCTAGACTACAATGATCCATAAATTAAAAAAC |
| Primers used in yeast two-hybrid | BD-CbuSPL9F | CATATGATGGAAAAGGGTTCTTCCTCCT |
|  | BD-CbuSPL9R | GTCGACGTCAGATAGAGGATTTTGTGAGTTTCCC |
|  | AD-CbuHMGAF | CATATGATGGCGAGCGAAGAAGTACA |
|  | AD-CbuHMGAR | GTCGACGCTACAATGATCCATAAATTAAAAAAC |
| Primers used in  prokaryoticexpression | CbuSPL9-6P-F | GGATCCATGGAAAAGGGTTCTTCCTC |
|  | CbuSPL9-6P-R | CTCGAGTCAGATAGAGGATTTTGTGAGTTT |
|  | CbuHMGA-HIS-F | CGCGGATCCATGGCGAGCGAAGAAGTACAG |
|  | CbuHMGA-HIS-R | CGCAAGCTTCTAAGCCCCTACGGGTGCGGC |
| Primers used in BiFC | CbuSPL9F | CACCATGGAAAAGGGTTCTTCCTCCTC |
|  | CbuSPL9R | AAGTGACCAGTGCACAGAAGAATC |
|  | CbuHMGAF | CACCATGGCGAGCGAAGAAGTACAGG |
|  | CbuHMGAR | AGCCCCTACGGGTGCGG |
|  | M13F | GTTGTAAAACGACGGCCAG |
|  | M13R | CAGGAAACAGCTATGAC |
|  | CaMV35S-F | GACGCACAATCCCACTATCC |
| Primers used in qRT-PCR | U6F | CTCGCTTCGGCAGCACATAT |
|  | miR156 | GCGTTGACAGAAGATAGAGAGCAC |
|  | CbuAP1-F | TGAGCCTCAATCACCTGCAAA |
|  | CbuAP1-R | GCTCTTGTATGGCCCTTTCCT |
|  | CbuLeafy-F | ACACCACACATGCCCTTGAT |
|  | CbuLeafy-R | CTCCGCCTCCACAATTCTCA |
