# Supplementary figures and images for: Potential function of CbuSPL and gene encoding its interacting protein during flowering in Catalpa bungei
Source: BMC Plant Biol. 2020 Mar 6;20:105. doi: 10.1186/s12870-020-2303-z (PMC7060540; doi:10.1186/s12870-020-2303-z)

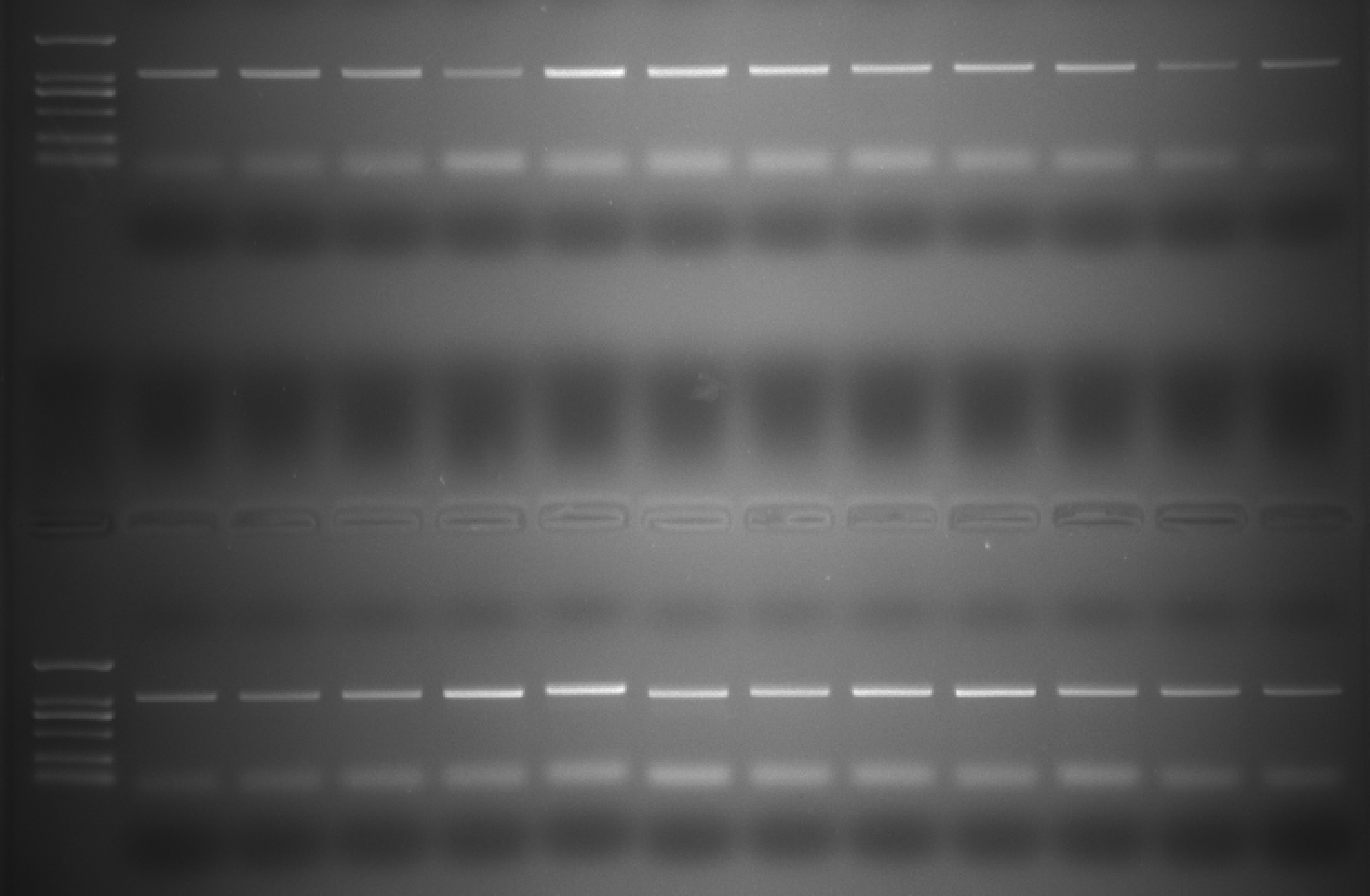

Supplement: Supplementary file 2 — Additional file 2: Figure S1. Verification of oe-SPL9 positive Arabidopsis. [file 12870_2020_2303_MOESM2_ESM.jpg]

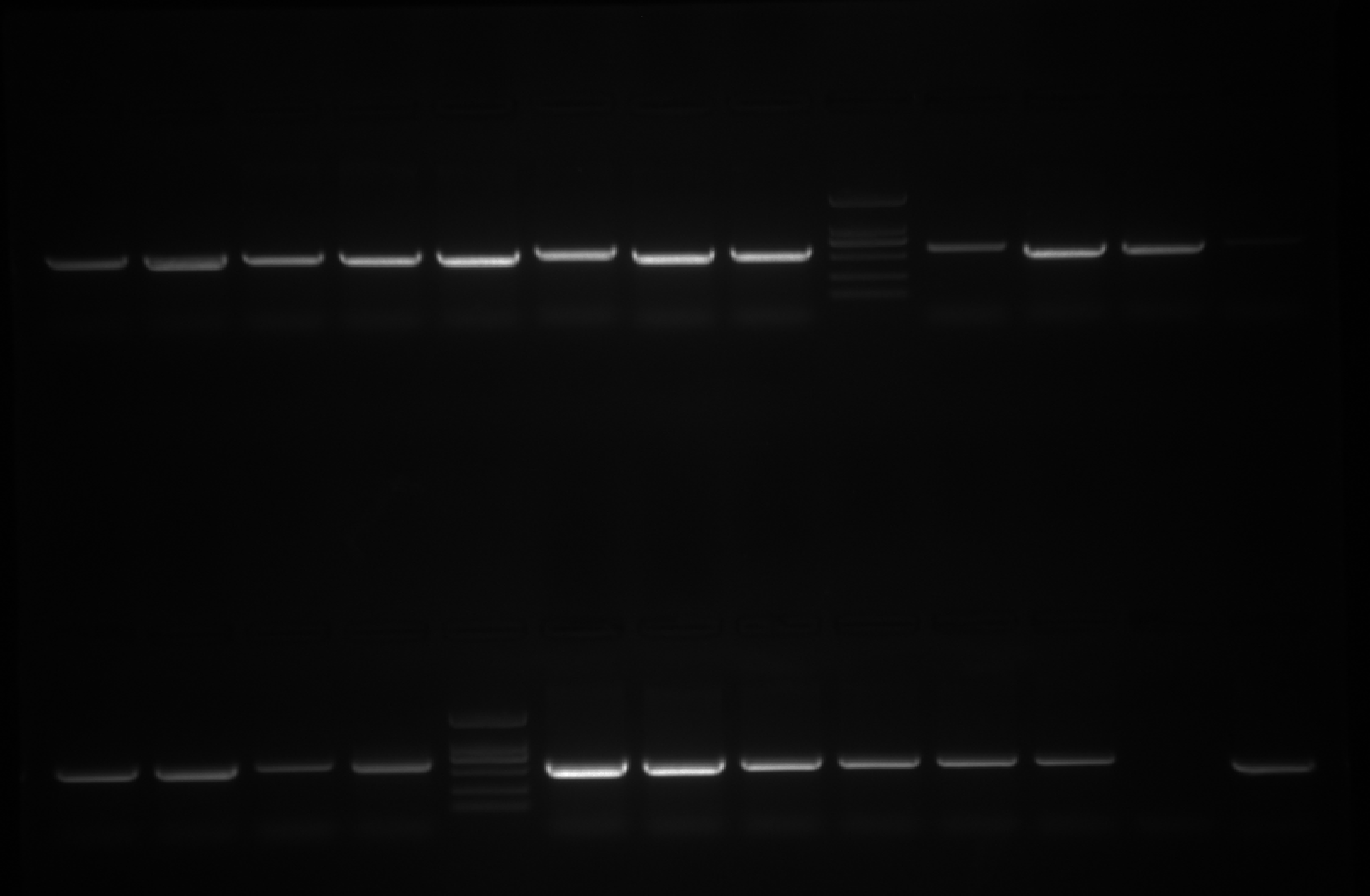

Supplement: Supplementary file 3 — Additional file 3: Figure S2. Verification of oe-HMGA positive Arabidopsis. [file 12870_2020_2303_MOESM3_ESM.jpg]

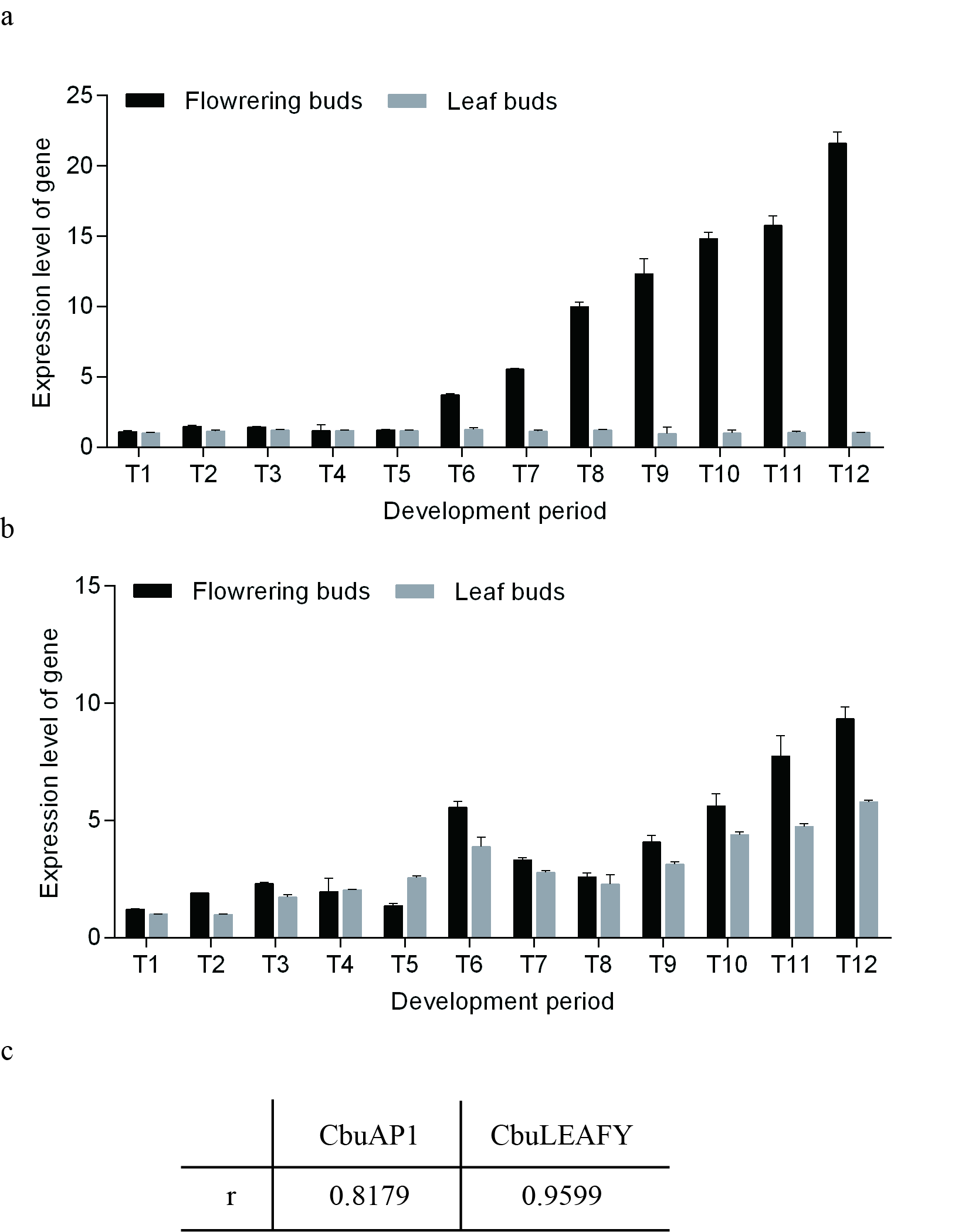

Supplement: Supplementary file 7 — Additional file 7: Figure S4. Expression profile of CbuLeafy and CbuAP1 in the flower buds and leaf buds during the developmental periods of C. bungei. [file 12870_2020_2303_MOESM7_ESM.tif]
