# Supplementary material for: Potential function of CbuSPL and gene encoding its interacting protein during flowering in Catalpa bungei
Source: BMC Plant Biol. 2020 Mar 6;20:105. doi: 10.1186/s12870-020-2303-z (PMC7060540; doi:10.1186/s12870-020-2303-z)
Supplement: Supplementary file 4 — Additional file 4: Table S2. Details of motif-sequences of CbuSPL9 were identified by MEME. [file 12870_2020_2303_MOESM4_ESM.docx]

**Table S2 Details of motif-sequences of CbuSPL9 were identified by MEME**

| No. | Sequence（5’-3’） | Quantity of motif site |
| --- | --- | --- |
| 1 | YYCRHKVCGMHSKSPKVIVAGLEQRFCQQCSRFHQLPEFDQGKRSCRRRL | 16 |
| 2 | RCQVEGCKVDLSDAK | 16 |
| 3 | AGHNERRRKP | 16 |
| 4 | RTGRIVFKLFGKEPNEFPIVLRGQILDWLSHSPTDMESYIRPGCIVLTIY | 4 |
| 5 | PAGLTPLHIAAGKDGSEDVLDALTEDPAMVGIEAWKTCRDSTGFTPEDYA | 3 |
| 6 | LGLKLGKRTYFEDFW | 7 |
| 7 | AYRPAMLSMVAIAAVCVCVALLFKSCPEVLYVFQ | 3 |
| 8 | FFPFLVVEDDDVCSEIRILETTLEFTGTDSAKQAMDFIHEIGWLLHR | 2 |
| 9 | FPLIRFQWLIEFSMDREWCAVIRKLLNMFFD | 3 |
| 10 | QAETAWEELSDDLGFSLGKLLDLSDDPLWTTGWIYVRVQNQLAFVYNGQV | 3 |
| 11 | GKRSVEWDLNDWKWD | 5 |
| 12 | ATLSELCLLHRAVRKNSKPMVEMLLRY | 5 |
| 13 | EKAQFTVKGMNLRQRGTRLLCSVEGKYLIQETT | 4 |
| 14 | GRDSWPNTTSERGLGNQSATTGKYQLPYQGNSQNP | 2 |
| 15 | DHHHQSRRQYMEDENTRAYDSSSHHTNW | 2 |
| 16 | VNFSCDMPILSGRGFMEIEDQ | 2 |
| 17 | FDNHSRSGGFMMDFSAY | 2 |
| 18 | MECNAKPPFQWELENLISFGTSTAEVPRKLKPMEWEI | 2 |
| 19 | DSNCALSLLSN | 7 |
| 20 | DDQTSNYMLITLLKILSNIHSNQSDQ | 2 |
