# Supplementary material for: Potential function of CbuSPL and gene encoding its interacting protein during flowering in Catalpa bungei
Source: BMC Plant Biol. 2020 Mar 6;20:105. doi: 10.1186/s12870-020-2303-z (PMC7060540; doi:10.1186/s12870-020-2303-z)
Supplement: Supplementary file 5 — Additional file 5: Tables S3. Details of CbuSPL9 was predicted to be a target of miR156. [file 12870_2020_2303_MOESM5_ESM.docx]

**Table S3 Details of *CbuSPL9* was predicted to be a target of miR156**

| miRNA | Target | miRNA start | miRNA end | Target start | Target end | miRNA aligned fragment | Target aligned fragment |
| --- | --- | --- | --- | --- | --- | --- | --- |
| ath-miR156i | CbuSPL9 | 1 | 20 | 761 | 780 | UGACAGAAGAGAGAGAGCAG | GUGCUCUCUCUCUUCUGUCA |
| ath-miR156j | CbuSPL9 | 1 | 20 | 761 | 780 | UGACAGAAGAGAGAGAGCAC | GUGCUCUCUCUCUUCUGUCA |
| ath-miR156a-5p | CbuSPL9 | 1 | 20 | 761 | 780 | UGACAGAAGAGAGUGAGCAC | GUGCUCUCUCUCUUCUGUCA |
| ath-miR156b-5p | CbuSPL9 | 1 | 20 | 761 | 780 | UGACAGAAGAGAGUGAGCAC | GUGCUCUCUCUCUUCUGUCA |
| ath-miR156c-5p | CbuSPL9 | 1 | 20 | 761 | 780 | UGACAGAAGAGAGUGAGCAC | GUGCUCUCUCUCUUCUGUCA |
| ath-miR156d-5p | CbuSPL9 | 1 | 20 | 761 | 780 | UGACAGAAGAGAGUGAGCAC | GUGCUCUCUCUCUUCUGUCA |
| ath-miR156e | CbuSPL9 | 1 | 20 | 761 | 780 | UGACAGAAGAGAGUGAGCAC | GUGCUCUCUCUCUUCUGUCA |
| ath-miR156f-5p | CbuSPL9 | 1 | 20 | 761 | 780 | UGACAGAAGAGAGUGAGCAC | GUGCUCUCUCUCUUCUGUCA |
| ath-miR156h | CbuSPL9 | 1 | 20 | 761 | 780 | UGACAGAAGAAAGAGAGCAC | GUGCUCUCUCUCUUCUGUCA |
