# Supplementary material for: Potential function of CbuSPL and gene encoding its interacting protein during flowering in Catalpa bungei
Source: BMC Plant Biol. 2020 Mar 6;20:105. doi: 10.1186/s12870-020-2303-z (PMC7060540; doi:10.1186/s12870-020-2303-z)
Supplement: Supplementary file 8 — Additional file 8: Table S4. Statistics of mutant of floral organs in oe-SPL9 Arabidopsis. [file 12870_2020_2303_MOESM8_ESM.docx]

**Table S4 Statistics of mutant of floral organs in *oe-SPL9* Arabidopsis**

| Type | overlapping petals | Change in petals number | shrunken petals |
| --- | --- | --- | --- |
| CbuSPL9-1 | - | * | * |
| CbuSPL9-2 | - | * | - |
| CbuSPL9-3 | - | - | - |
| CbuSPL9-4 | * | - | - |
| CbuSPL9-5 | - | * | - |
| CbuSPL9-6 | * | - | - |
| CbuSPL9-7 | * | * | - |
| CbuSPL9-8 | - | * | - |
| CbuSPL9-9 | - | - | - |
| CbuSPL9-10 | - | - | * |
| CbuSPL9-11 | * | * | - |
| CbuSPL9-12 | - | * | * |
| CbuSPL9-13 | * | * | - |
| CbuSPL9-14 | * | * | - |
| CbuSPL9-15 | - | - | * |
| CbuSPL9-16 | - | - | * |
| CbuSPL9-17 | * | * | - |
| CbuSPL9-18 | * | - | - |
| CbuSPL9-19 | - | * | - |
| CbuSPL9-20 | - | * |  |
| CbuSPL9-21 | - | - | * |
| CbuSPL9-22 | - | - | - |
| CbuSPL9-23 | - | * | - |
| CbuSPL9-24 | - | - | * |
| CbuSPL9-25 | - | * | - |
| CbuSPL9-26 | - | * | * |
| CbuSPL9-27 | * | - | - |
| CbuSPL9-28 | - | * | - |
| CbuSPL9-29 | * | * | - |
| CbuSPL9-30 | - | * | - |
| CbuSPL9-31 | - | - | * |
| CbuSPL9-32 | * | - | - |
| CbuSPL9-33 | - | * | - |
| CbuSPL9-34 | - | - | * |
| CbuSPL9-35 | * | * | - |
| CbuSPL9-36 | - | * | - |
