# Supplementary material for: Potential function of CbuSPL and gene encoding its interacting protein during flowering in Catalpa bungei
Source: BMC Plant Biol. 2020 Mar 6;20:105. doi: 10.1186/s12870-020-2303-z (PMC7060540; doi:10.1186/s12870-020-2303-z)
Supplement: Supplementary file 9 — Additional file 9: Table S5. Statistics of mutant of flowering time in oe-SPL9 Arabidopsis. [file 12870_2020_2303_MOESM9_ESM.docx]

**Table S5 Statistics of mutant of flowering time in *oe-SPL9* Arabidopsis**

| Type | DAY | Type | DAY |
| --- | --- | --- | --- |
| CbuSPL9-01 | 22 | WT-01 | 27 |
| CbuSPL9-02 | 17 | WT-02 | 27 |
| CbuSPL9-03 | 23 | WT-03 | 26 |
| CbuSPL9-04 | 21 | WT-04 | 27 |
| CbuSPL9-05 | 22 | WT-05 | 25 |
| CbuSPL9-06 | 16 | WT-06 | 28 |
| CbuSPL9-07 | 22 | WT-07 | 28 |
| CbuSPL9-08 | 23 | WT-08 | 26 |
| CbuSPL9-09 | 23 | WT-09 | 25 |
| CbuSPL9-10 | 22 | WT-10 | 27 |
| CbuSPL9-11 | 21 | WT-11 | 25 |
| CbuSPL9-12 | 22 | WT-12 | 27 |
| CbuSPL9-13 | 23 | WT-13 | 27 |
| CbuSPL9-14 | 22 | WT-14 | 26 |
| CbuSPL9-15 | 22 | WT-15 | 27 |
| CbuSPL9-16 | 24 | WT-16 | 27 |
| CbuSPL9-17 | 22 | WT-17 | 25 |
| CbuSPL9-18 | 18 | WT-18 | 29 |
| CbuSPL9-19 | 23 | WT-19 | 27 |
| CbuSPL9-20 | 21 | WT-20 | 28 |
| CbuSPL9-21 | 24 | WT-21 | 27 |
| CbuSPL9-22 | 23 | WT-22 | 26 |
| CbuSPL9-23 | 21 | WT-23 | 26 |
| CbuSPL9-24 | 24 | WT-24 | 27 |
| CbuSPL9-25 | 21 | WT-25 | 27 |
| CbuSPL9-26 | 23 | WT-26 | 25 |
| CbuSPL9-27 | 21 | WT-27 | 26 |
| CbuSPL9-28 | 18 | WT-28 | 26 |
| CbuSPL9-29 | 21 | WT-29 | 27 |
| CbuSPL9-30 | 20 | WT-30 | 26 |
