# Supplementary material for: Potential function of CbuSPL and gene encoding its interacting protein during flowering in Catalpa bungei
Source: BMC Plant Biol. 2020 Mar 6;20:105. doi: 10.1186/s12870-020-2303-z (PMC7060540; doi:10.1186/s12870-020-2303-z)
Supplement: Supplementary file 10 — Additional file 10: Table S6. Statistics of mutant of floral organs in oe-HMGA Arabidopsis. [file 12870_2020_2303_MOESM10_ESM.docx]

**Table S6 Statistics of mutant of floral organs in *oe-HMGA*Arabidopsis.**

| Type | Overlapping petals | Change in petals number | shrunken petals |
| --- | --- | --- | --- |
| CbuHMGA-1 | * | * |  |
| CbuHMGA-2 |  |  | * |
| CbuHMGA-3 |  | * |  |
| CbuHMGA-4 | - | - | - |
| CbuHMGA-5 |  | * | * |
| CbuHMGA-6 |  | * |  |
| CbuHMGA-7 | * | * |  |
| CbuHMGA-8 |  | * |  |
| CbuHMGA-9 |  |  | * |
| CbuHMGA-10 | * | * |  |
| CbuHMGA-11 | - | - | - |
| CbuHMGA-12 | * | * |  |
| CbuHMGA-13 |  | * |  |
| CbuHMGA-14 |  | * |  |
| CbuHMGA-15 |  |  | * |
| CbuHMGA-16 |  | * |  |
| CbuHMGA-17 |  | * | * |
| CbuHMGA-18 |  | * |  |
| CbuHMGA-19 | - | - | - |
| CbuHMGA-20 | - | - | - |
| CbuHMGA-21 |  | * |  |
| CbuHMGA-22 | * |  |  |
| CbuHMGA-23 |  |  | * |
| CbuHMGA-24 |  | * |  |
| CbuHMGA-25 |  |  | * |
| CbuHMGA-26 | * | * |  |
| CbuHMGA-27 |  |  | * |
| CbuHMGA-28 | - | - | - |
| CbuHMGA-29 |  |  | * |
| CbuHMGA-30 | * | * |  |
| CbuHMGA-31 |  |  | * |
| CbuHMGA-32 |  | * |  |
| CbuHMGA-33 |  |  | * |
| CbuHMGA-34 |  | * |  |
| CbuHMGA-35 | - | - | - |
| CbuHMGA-36 | * | * |  |
