# Supplementary material for: Potential function of CbuSPL and gene encoding its interacting protein during flowering in Catalpa bungei
Source: BMC Plant Biol. 2020 Mar 6;20:105. doi: 10.1186/s12870-020-2303-z (PMC7060540; doi:10.1186/s12870-020-2303-z)
Supplement: Supplementary file 11 — Additional file 11: Table S7. Statistics of mutant of flowering time in oe-HMGA Arabidopsis. [file 12870_2020_2303_MOESM11_ESM.docx]

**Table S7 Statistics of mutant of flowering time in *oe-HMGA*Arabidopsis.**

| Type | Time | Type | Time |
| --- | --- | --- | --- |
| CbuHMGA-01 | 25 | WT-01 | 27 |
| CbuHMGA-02 | 25 | WT-02 | 27 |
| CbuHMGA-03 | 26 | WT-03 | 26 |
| CbuHMGA-04 | 24 | WT-04 | 27 |
| CbuHMGA-05 | 27 | WT-05 | 25 |
| CbuHMGA-06 | 28 | WT-06 | 28 |
| CbuHMGA-07 | 26 | WT-07 | 28 |
| CbuHMGA-08 | 24 | WT-08 | 26 |
| CbuHMGA-09 | 28 | WT-09 | 25 |
| CbuHMGA-10 | 27 | WT-10 | 27 |
| CbuHMGA-11 | 24 | WT-11 | 25 |
| CbuHMGA-12 | 28 | WT-12 | 27 |
| CbuHMGA-13 | 27 | WT-13 | 27 |
| CbuHMGA-14 | 26 | WT-14 | 26 |
| CbuHMGA-15 | 25 | WT-15 | 27 |
| CbuHMGA-16 | 28 | WT-16 | 27 |
| CbuHMGA-17 | 25 | WT-17 | 25 |
| CbuHMGA-18 | 24 | WT-18 | 29 |
| CbuHMGA-19 | 28 | WT-19 | 27 |
| CbuHMGA-20 | 27 | WT-20 | 28 |
| CbuHMGA-21 | 26 | WT-21 | 27 |
| CbuHMGA-22 | 26 | WT-22 | 26 |
| CbuHMGA-23 | 26 | WT-23 | 26 |
| CbuHMGA-24 | 25 | WT-24 | 27 |
| CbuHMGA-25 | 27 | WT-25 | 27 |
| CbuHMGA-26 | 25 | WT-26 | 25 |
| CbuHMGA-27 | 27 | WT-27 | 26 |
| CbuHMGA-28 | 26 | WT-28 | 26 |
| CbuHMGA-29 | 26 | WT-29 | 27 |
| CbuHMGA-30 | 28 | WT-30 | 26 |
